# Improvements in Mechanical and Shape-Memory Properties of Bio-Based Composite: Effects of Adding Carbon Fiber and Graphene Nanoparticles

**DOI:** 10.3390/polym15234513

**Published:** 2023-11-24

**Authors:** Panuwat Luengrojanakul, Phattharin Mora, Kittipon Bunyanuwat, Chanchira Jubsilp, Sarawut Rimdusit

**Affiliations:** 1 Center of Excellence in Polymeric Materials for Medical Practice Devices, Department of Chemical Engineering, Faculty of Engineering, Chulalongkorn University, Bangkok 10330, Thailand; panuwat.lu@gmail.com (P.L.); golf_1990@hotmail.com (K.B.); 2 Department of Chemical Engineering, Faculty of Engineering, Srinakharinwirot University, Nakhonnayok 26120, Thailand; phattarin@g.swu.ac.th (P.M.); chanchira@g.swu.ac.th (C.J.)

**Keywords:** shape-memory polymer composite, graphene, thermosetting resin, mechanical properties

## Abstract

Shape-memory carbon fiber (CF) polymer composites reinforced with graphene nanoplatelets (GnPs) as a filler based on a bio-based V-fa/ECO copolymer were prepared at different graphene GnPs and CF mass fractions using the hand lay-up and hot-pressing methods. The obtained composite specimens were subjected to flexural, dynamic mechanical, and shape-memory analyses. The obtained results revealed that the flexural strength and modulus were improved by the addition of the GnPs and CF due to the improvement in the interfacial adhesion and fiber reinforcement with up to 3 wt.% GnPs and 60 wt.% CF. Additionally, appreciable improvements in the shape-memory performance were achieved with the addition of the GnPs, where values of up to 93% and 96% were recorded for the shape fixity and recovery, respectively. The shape-memory performance was affected by the fiber mass fraction, with the composites retaining the shape-memory effect albeit with a significant drop in performance at higher fiber mass fractions. Lastly, the specimens at 40 wt.% CF and 3 wt.% GnPs were determined to be the optimum compositions for the best performance of the bio-based SMP composite.

## 1. Introduction

Shape-memory polymers (SMPs) are a class of advanced stimuli-responsive polymers that, when exposed to external stimuli, possesses the ability to change into a temporary shape after fabrication and revert back to their original permanent shape, and this effect is commonly activated by heat. The structures of typical SMPs are composed of two parts: a reversible phase and a stationary phase, which can be based on either physical or chemical changes. The reversible phase usually has a structure of a flexible linear chain and is responsible for allowing the deformation of the shape. On the other hand, the stationary phase, often a stable covalent network such as an aromatic structure, is responsible for maintaining the original shape [1,2,3]. The shape-altering capability of SMPs render this class of material as being highly applicable in various fields, including automotives, biomedical, energy storage, smart textiles, and structural applications. The utilization of SMPs could confer additional advantages compared to conventional polymers.

Still, SMPs have drawbacks that have to do with their low strength, stiffness, and thermal conductivity, thus limiting their potential applications. Nevertheless, it has been established that reinforcing the SMP matrix with a fiber could improve the mechanical properties and enhance the recovery stress of SMP composites, which is a crucial parameter in SMP systems [4,5,6]. As such, SMPs have been gaining increasing popularity as promising candidates in the production of high-performance composites in the past decades. Carbon fiber has been extensively used in the reinforcement of SMP systems aimed at structural applications due to their light weight relative to their superior mechanical properties, their high chemical resistance, and their high thermal stability. In addition, carbon fiber could also provide a conductive path to achieve the Joule heating effect, as demonstrated by Lu et al. [7], providing an alternative method of actuation via electricity.

However, the reinforcement of SMPs with fiber has been reported to negatively affect the shape-memory performance. For instance, Nishikawa et al. [8] reported that the addition of a high fiber volume fraction is accompanied by a decrease in the shape fixity ratio. Similarly, the study by Fejős et al. [9] reported that the shape fixity and recovery rate were negatively impacted, but the recovery stress was enhanced because of the woven glass fiber reinforcement (37 vol.%). In another study, Xu et al. [10] found that the stacking orientation of unidirectional carbon fiber has an effect on the shape-memory performance of the composite. Therefore, the fiber and matrix content should be balanced when considering the mechanical and shape-memory properties. The addition of nanomaterials such as montmorillonite (MMT), carbon nanotubes (CNT), and graphene-based fillers has also been studied [11,12,13,14,15]. For instance, Gangineni et al. [14] prepared a plain-weaved carbon fiber–epoxy composite decorated with a graphene-based filler, and their results revealed that the load-bearing capacity of the composite was increased by up to 9.6%. Also, Li et al. [16] prepared a shape-memory composite using hybrid reinforcement using graphite oxide (GO) and carbon fiber (CF), and their findings revealed an appreciable increase in both the shape fixity and shape recovery ratio.

Recently, many efforts have been put into developing thermosetting SMPs based on polybenzoxazines due to their excellent dimensional stability, molecular design diversity, typically high thermal stability, and the fact that they can be prepared from either petroleum- or bio-based chemicals. Furthermore, high-performing SMPs based on polybenzoxazine alloyed with other polymers (i.e., epoxy, urethane, polycaprolactone (PCL)) [17,18,19,20,21] using a long-chain aliphatic diamine as the reactant [22,23,24] or by manipulating the hydrogen bonding of neat polybenzoxazine [25] have been achieved. Furthermore, considering the concept of a bio-circular economy, sustainability, and eco-friendly production, bio-based shape-memory copolymers from polybenzoxazine based on vanillin-furfurylamine benzoxazine (V-fa) and epoxidized castor oil (ECO) was investigated by Amornkitbamrung et al. [18] and Hombunma et al. [20], and the independent results recorded showed that a shape recovery of 100% with a near-infrared stimulus of 96% with temperature stimulus were achieved for 50/50 wt.% V-fa/ECO, respectively. These studies revealed that the bio-based system (V-fa/ECO) exhibited excellent performance, and further improvements in terms of the mechanical and shape-memory properties could result in the production of a robust SMP composite material with possible applications in the production of automotive parts. Hitherto, this reliable system (V-fa/ECO) with reinforcement with CF and graphene nanoplatelets (GnPs) as fillers has not been investigated to the best of our knowledge.

Therefore, in this present study, a bio-based SMP composite using V-fa/ECO at 50/50 wt.% was prepared by the hand lay-up method followed by the hot-pressing molding method. Furthermore, detailed characterizations were carried out, which showed that significant improvements in terms of the mechanical and shape-memory properties of the novel composite were achieved.

## 2. Materials and Methods

### 2.1. Materials

Vanillin (98%) and furfurylamine (98%) were obtained from TCI America (Portland, OR, USA). Paraformaldehyde (AR grade) was obtained from Merck Co., Ltd. (Darmstadt, Germany). Epoxidized castor oil (ECO) was kindly provided by Aditya Birla Chemicals Ltd. (Rayong, Thailand). Graphene nanoplatelets (xGnP^®^) (H-15 grade) were purchased from XG Sciences (Lansing, MI, USA). Plain-weaved carbon fiber (CF) (TI-6101) fabric was obtained from Entra Korea (Busan, Republic of Korea). All the chemicals were used as received.

### 2.2. Synthesis of Benzoxazine Monomer

Bio-based benzoxazine monomer based on vanillin and furfurylamine (V-fa) was synthesized using a solventless procedure. Vanillin, furfurylamine, and paraformaldehyde were mixed together at a 1:1:2 molar ratio at 110 °C under constant agitation for 1 h. The obtained product was a transparent yellow viscous solid at room temperature and was used without further purification.

### 2.3. Preparation of Shape-Memory Carbon-Fiber-Reinforced Composite and Neat V-fa/ECO Copolymer

The obtained graphene nanoplatelets (GnPs) and ECO were dried at 80 °C overnight in a convection oven and kept in a desiccator before usage. The carbon-fiber-reinforced polymer composite (CFRP) was prepared by the hand lay-up method followed by hot pressing. The carbon fiber (CF) was laid-up in an aluminum mold at varying fiber mass fractions (i.e., 40%, 50%, 60%, 70%). Then, the resin mixture was prepared by heating a 50/50 wt.% mixture of the synthesized V-fa and the dried ECO to 90 °C for 15 min until a homogenous mixture was obtained. Next, the GnPs were then introduced into the resin mixture at varying amounts (i.e., 0, 1, 3, and 5 wt.%) and stirred for another 30 min. The compound was coated on the fabric at 90 °C to afford prepregs. The prepregs were then stacked together until a thickness of approximately 2 mm was obtained, and they were then kept in an air-circulating oven at a constant temperature of 140 °C for 3 h to partially cure. Finally, the respective formulations were transferred to a compression molder and kept at temperatures of 150 °C for 1 h, 160 °C for 1 h, 170 °C for 2 h, and 180 °C for 2 h at a pressure of 50 bar. Similarly, neat V-fa/ECO was prepared following the same curing and hot-pressing procedure used for the composite fabrication.

### 2.4. Characterizations of the Shape-Memory Carbon-Fiber-Reinforced Composite

The flexural properties of the composite samples and neat V-fa/ECO were examined according to ASTM D790 [26] using a universal testing machine (Instron 5567, Norwood, MA, USA, 1 kN load cell) under a three-point bending mode with the strain measured using crosshead displacement. The tests were performed with specimen dimensions of 12.7 mm × 50 mm × 2 mm. An average of at least five specimens were used for the experiment. The crosshead speed and span-to-depth ratio used for the composite specimens were 1 mm/min and 16:1, respectively. For the neat V-fa/ECO, a higher crosshead speed of 8.5 mm/min was used instead. The dynamic mechanical thermal analysis of the composite specimens was also carried out using a dynamic mechanical analyzer (model DMA1, Mettler-Toledo, Greifensee, Switzerland). The analysis was conducted from −100–200 °C at a heating rate of 2 °C/min with a frequency of 1 Hz under atmospheric air. The test was performed under the three-point bending mode with a preload force of 2 N and with specimen dimensions of 10 mm × 45 mm × 2 mm. The glass transition temperature (T_g_) was reported in terms of the maxima of the loss tangent curve (tan δ). A scanning electron microscope (JEOL JSM-6510A, Tokyo, Japan) was used to assess the flexural fracture surfaces of the composite specimens using an acceleration voltage 5 kV. All specimens were sputtered with gold before observation.

The shape-memory properties of the composites were investigated via hot deformation using the same specimen dimensions as the flexural test. The shape-memory process was accomplished using a universal testing machine environmental chamber by first heating the specimens in the thermal chamber to a temperature of T_g_ + 20 °C at a heating rate of 5 °C/min and then deforming them to a 5% strain under a crosshead speed of 1 mm/min and a span length of 32 mm. In the second step, the specimens were cooled down to room temperature, and the crosshead was removed thereafter. In the third step, the specimens were once again reheated to the same temperature used for the deformation to initiate the shape recovery; the strains were measured after 20 min. The shape fixity (*R_f_*) and shape recovery (*R_r_*) ratios of each specimen were calculated according to the following equations:

(1) $$R_{f}=\frac{\varepsilon_{1}}{\varepsilon_{max}}\times 100\%$$

(2) $$R_{r}=\frac{\varepsilon_{1}-\varepsilon_{2}}{\varepsilon_{1}}\times 100\%$$

where $\varepsilon_{max}$ is the maximum strain from deformation; $\varepsilon_{1}$ is the fixed strain after cooling down with the external stress removed; and $\varepsilon_{2}$ is the residual unrecovered strain. The recovery stress was investigated under constrained recovery, in which the stress was recorded during the recovery process. The constrained recovery process was initiated by maintaining the crosshead position after shape fixation to keep the deformation constant while heating the specimens in a thermal chamber at a heating rate of 5 °C/min to T_g_ + 20 °C, and this temperature was maintained afterwards. All specimens were cut into the required shape for characterization using a diamond saw.

## 3. Results and Discussion

### 3.1. Flexural Properties of CFRP

**Figure 1:**
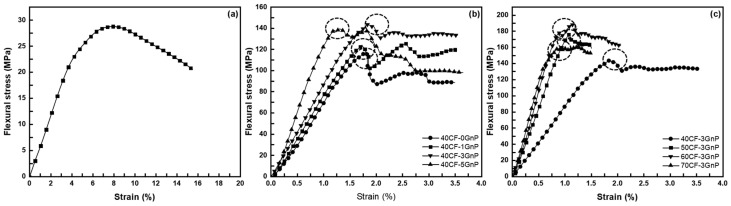
Representative flexural stress–strain curve of (**a**) neat V-fa/ECO (50/50 wt.%), and V-fa/ECO shape-memory composite: (**b**) 40 wt.% CF at varying GnPs contents; (**c**) 3 wt.% GnPs at varying CF mass fractions.

Figure 1a shows the representative flexural stress–strain curve of the neat V-fa/ECO. As can be seen, the yield point of the copolymer occurred at a very high strain percentage without the specimen breaking due to the flexible and plasticizing nature of ECO. Figure 1b shows the fiber-reinforced composites against various GnPs contents at 40 wt.% CF. An increase in both the flexural strength and modulus was identified with the fiber reinforcement and increasing GnPs.

As can be observed in Table 1, the flexural strength and modulus of the neat V-fa/ECO were determined to be 27.69 MPa and 0.55 GPa, respectively. Meanwhile, the flexural strength and modulus of the unfilled composite stood at 117.5 MPa and 10.3 GPa, respectively, showing a significant increase compared to the unreinforced V-fa/ECO. The mechanical properties of the composites reached the maximum when 3 wt.% GnPs was added, where a 16.8% and 16.2% enhancement in the flexural strength and modulus were recorded, respectively. On the whole, the curves gradually dropped after reaching the maximum stress for the specimens with 1 wt.% and 3 wt.% GnPs as opposed to the unfilled and 5 wt.% GnPs specimens, where a more sudden and significant drop in the curve can be detected. A gradual drop implies that the crack propagation proceeds in a stable manner, whereas a sudden drop signifies catastrophic failure. The improvement in the mechanical properties was attributed to the proposed π-π interactions that occur between V-fa/ECO copolymers in their solid state [18]. The aromatic structure of the V-fa could then form π-π interactions with both the carbon fiber and GnPs, promoting stronger interfacial adhesion between the fiber and matrix. The aforementioned explanation was responsible for the enhanced interfacial adhesion, which in turn led to improved mechanical performance by facilitating effective stress transfer throughout the composite. However, increasing the amount of GnPs to 5 wt.% negatively impacted the properties instead due to excessive agglomeration of the nanoplatelets, perhaps due to the agglomerated particles acting as stress concentrators that could result in premature failure from internal delamination [12,27]. In addition, it was evident from the stress–strain curve that the 5 wt.% GnPs specimens experienced a premature ply failure at lower deflection (encircled region) when compared to the specimens with other compositions. Nonetheless, it is worth noting that this agglomeration could still provide a degree of mechanical reinforcement when compared to the unfilled composite specimens.

Therefore, the CF composite filled with 3 wt.% GnPs was chosen for further characterization. According to Figure 1c, the flexural strength and modulus increased progressively with increasing the fiber mass fraction up to 60 wt.% CF, where it then decreased. At a higher fiber mass fraction, the fibers were not sufficiently wetted, and the polymer matrix responsible for the bonding was reduced. As a consequence, delamination occurred due to fiber–matrix debonding in the case of the 70 wt.% CF specimen. The decrease in the interlamellar bonding properties could be explained by the fibers compressing against each other, obstructing efficient stress transfer [28].

### 3.2. Dynamic Mechanical Properties of CFRP

**Figure 2:**
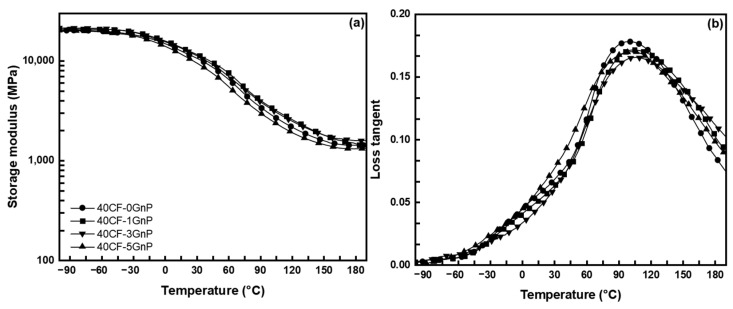
(**a**) Storage modulus and (**b**) loss tangent curves of V-fa/ECO at 40 wt.% CF and varying GnPs content.

The dynamic mechanical properties of the CFRP at 40 wt.% CF and 0–5 wt.% GnPs loading are depicted in Figure 2. Figure 2a presents the storage modulus as a function of temperature. As can be seen, as the temperature increased, the polymer matrix softened and its ability to absorb energy decreased, causing a decrease in the storage modulus value. By incorporating the GnPs along with the CF, the storage modulus of the polymer matrix slightly improved throughout the whole temperature range. The glassy state storage modulus reached the maximum at 3 wt.% GnPs with a value of 21.4 GPa (Table 2). The reinforcing fiber and wrinkled structure of the GnPs obstructed the polymer chain mobility and stiffening of the composites and improved the thermal stability, contributing to the improvement in the overall dynamic mechanical properties [29].

Figure 2b shows the loss tangent curve as a function of temperature. As can be seen, the loss tangent showed asymmetry in the curve, indicating a certain degree of non-homogeneity in the crosslinked network of the cured polymer matrix, with the ECO-rich domain being responsible for the low-temperature side and the apparent peak towards the high-temperature side corresponding to the V-fa-rich domain. Similarly, the value of T_g_, as determined by the peak maxima of the loss tangent curve, was found to shift to a higher value up to 104 °C with increasing the GnPs content. Moreover, the loss tangent curve or damping factor is the ratio of the loss to the storage modulus and is associated with the segmental mobility chain as well as the degree of energy dissipation. Therefore, a lower loss tangent curve intensity implies less dissipation and, hence, better fiber–matrix adhesion [30]. Here, it can be seen that the loss tangent intensity was lowered with the addition of GnPs and was lowest at 3 wt.%, thus confirming the improved interfacial bonding. Further increment in the GnPs content instead resulted in decrements in the properties for both the storage modulus and T_g_, with these dropping down to 20.3 GPa and 103 °C (Table 2), respectively, along with a slight increase in loss tangent intensity. The composite stress transfer efficiency was reduced, but it was still higher than that of the neat composite. As observed, the storage modulus of the composites followed a similar trend to the static mechanical test.

**Figure 3:**
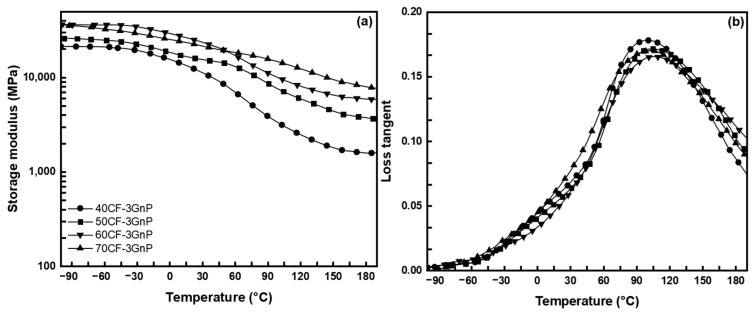
(**a**) Storage modulus and (**b**) loss tangent curves of V-fa/ECO at varying carbon fiber mass fraction and 3 wt.% GnPs.

The storage modulus and loss tangent of the 3 wt.% GnPs-filled CFRP at varying mass fiber fractions as a function of the temperature are displayed in Figure 3. The glassy state storage modulus and T_g_ increased with increasing the CF mass fraction, and the values ranged from 21.4 to 36.4 GPa (Table 2). However, the increase in T_g_ was remarkably high for the CFRP with a higher CF mass fraction (60–70 wt.%), which can be ascribed to insufficient resin preventing the full wetting of the fiber and the higher degree of phase separation between the ECO- and V-fa-rich domains. Meanwhile, the loss tangent intensity decreased with a higher fiber loading, as the polymer matrix is responsible for the energy dissipation, not the fiber. All the dynamic mechanical properties results are summarized in Table 2.

### 3.3. SEM Observations

**Figure 4:**
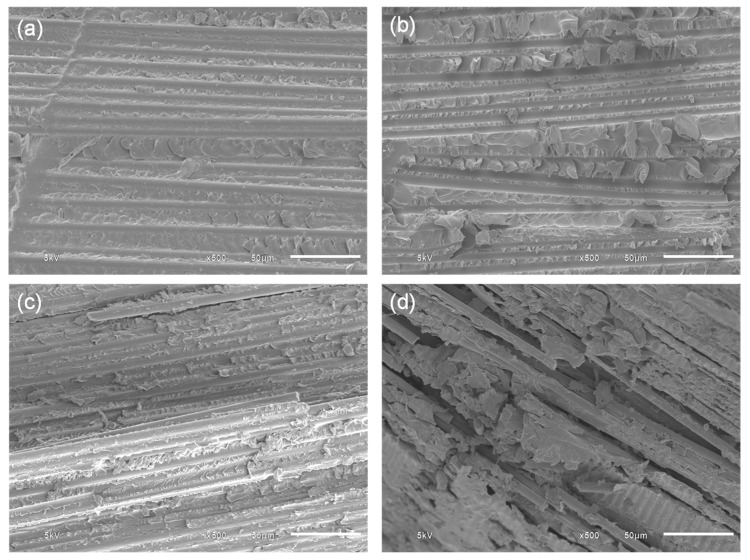
SEM micrographs of 40 wt.% CF CFRP after flexural testing: (**a**) neat, (**b**) 1 wt.% GnPs, (**c**) 3 wt.% GnPs, and (**d**) 5 wt.% GnPs.

**Figure 5:**
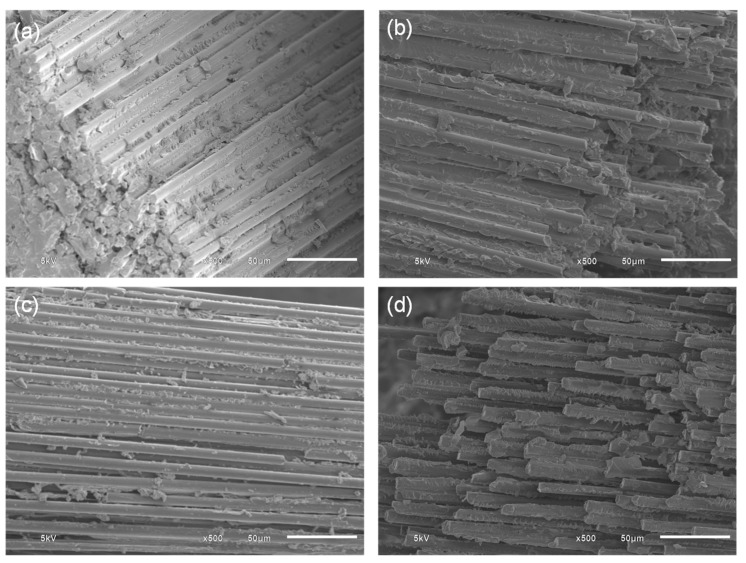
SEM micrographs of 3 wt.% GnPs filled CFRP at varying CF mass fractions after flexural testing: (**a**) 40 wt.%, (**b**) 50 wt.%, (**c**) 60 wt.%, and (**d**) 70 wt.%.

Figure 4a–c present the morphologies of the CFRPs, as obtained using a scanning electron microscope (SEM). It can be observed that the surface of filler-added composites exhibited a more irregular and rougher surface with increasing the GnPs content. The presence of the GnPs helped to play a role in reducing the stress concentration at the crack initiation site, allowing for higher amount of energy to be absorbed via plastic deformation [31]. On the other hand, agglomeration of the GnPs prevented the penetration of the resin and sufficient wetting of the fiber, as evidenced by the severe fiber–matrix debonding and fiber splitting, as shown in Figure 4d. Figure 5 shows the SEM micrographs of the flexural test specimens’ fracture surface at the broken fiber bundle region with varying fiber mass fractions. As can be seen, at a lower fiber mass fraction, the fiber bundle was still intact and a substantial amount of resin residue still adhered to the fiber surface, whereas the fiber bundles unbonded and the matrix completely detached at a higher fiber loading.

### 3.4. Shape-Memory Properties of CFRP

**Figure 6:**
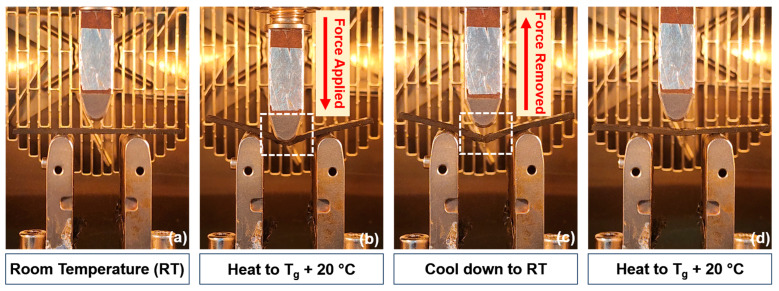
Shape-memory process of V-fa-/ECO-reinforced CFRP under thermal actuation: (**a**) original shape, (**b**) deformed temporary shape at T_g_ + 20 °C and 5% strain, (**c**) fixed temporary shape at room temperature, and (**d**) recovered original shape at T_g_ + 20 °C.

In the V-fa/ECO copolymer system, the rigid V-fa-rich domain acted as the stationary phase, which maintained the temporary shape, while the flexible aliphatic structure of the ECO acted as the reversible phase. As V-fa acted as the stationary phase, the composite was heated to a temperature above the value of T_g_ of the V-fa-rich domain, causing the copolymer to be in a rubbery state, and deformation of the composites became possible as the bonds between the matrix and fiber were weakened. After the deformation, cooling the composites down below T_g_ allowed the elastic stress to be frozen and the shape to be fixed. Heating the composites above T_g_ again caused the stress to be released, recovering its original shape. The unconstrained shape-memory process was conducted to evaluate the shape-memory properties in terms of the shape fixity and shape recovery. The shape fixation and unconstrained recovery processes are illustrated in Figure 6.

**Figure 7:**
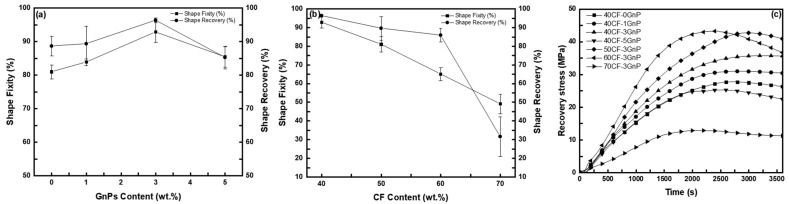
(**a**) Shape fixity and shape recovery ratios at T_g_ + 20 °C of wt.% CF shape-memory CFRP at 5% strain deformation at varying GnPs content, (**b**) shape fixity and shape recovery ratios at T_g_ + 20 °C of shape-memory CFRP at varying CF content and 3 wt.% GnPs, and (**c**) recovery stress versus time recorded by heating up to T_g_ + 20 °C.

Figure 7a displays the shape fixity and shape recovery ratio of the shape-memory CFRP with 40 wt.% CF at different GnPs contents. By comparison, the shape fixity and shape recovery ratio increased progressively with the increase in the GnPs content up to 3 wt.%, where the shape fixity and recovery ratio improved from 81% and 89% to 93% and 96%, respectively. Further addition to 5 wt.% caused the properties to deteriorate to 86% and 85% instead. In addition to allowing the stress to be uniformly dispersed, the improvement in the shape-memory properties could be attributed to the GnPs acting as an additional physical net point, preventing chain slippage [16,32]. However, at a higher CF mass fraction, the spring-back effect was especially prominent upon unloading due to the reduced resin matrix content. As spring-back does not represent the shape-memory performance, the effective shape recovery ratio consequently suffered. From Figure 7b, the shape-memory properties of the specimens with CF mass fractions higher than 50 wt.% saw a drastic decline, with the 70 wt.% CF specimens barely demonstrating any recovery at all. It was noted that the V-fa/ECO CFRP shape fixity was lower compared to their non-fiber-reinforced counterparts of ~85% [18,20] due to the higher resilience of the bidirectional carbon fiber, causing a loss in the shape-memory performance. The results obtained from the V-fa/ECO system in this study demonstrated a superior shape-memory performance than the anhydride-crosslinked bio-based epoxidized linseed oil fiber reinforced with continuous flax fiber reported by Fejős et al. [33], in which the shape recovery ratios only reached up to 70–80% at maximum.

The recovery stress was recorded under constant-strain conditions, where the specimen was heated and the generated force acted on the actuator, allowing the stress to be recorded. The stress evolution as a function of time during the constrained recovery is shown in Figure 7c, where a non-linear increase is obvious. The peak recovery stresses of the 40 wt.% CF were 27.7, 30.8, 35.7, and 25.2 MPa for the 0 to 5 wt.% GnPs specimens, respectively. Similarly, the peak recovery stresses of the specimens with varying fiber mass fractions were increased to 42.7 and 43.1 MPa for the 50 and 60 wt.% specimens, but they experienced a drastic drop to 12.8 MPa at 70 wt.%. At 70 wt.%, debonding between the fiber ply and the insufficient matrix responsible for the shape-memory effect could be the reason for the lower recovery stress. In general, the magnitude of the recovery stress increased with the GnPs content as well as the CF mass fraction. As has been previously mentioned, reinforcing the polymer matrix with the CF and GnPs resulted in an enhancement in the storage modulus. In turn, the specimen required more energy to be deformed, causing more energy to be frozen during the shape-fixing process [34]. The higher the frozen energy, the larger the exerted recovery force upon initiating the recovery process.

**Figure 8:**
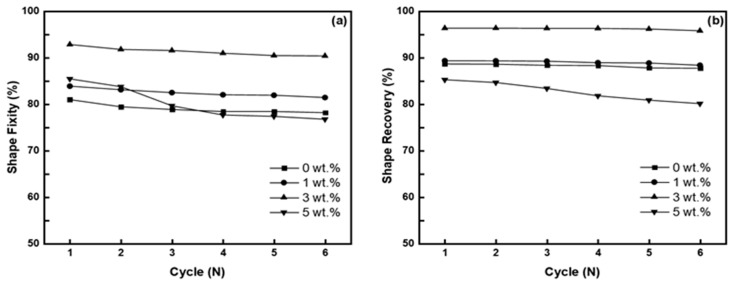
(**a**) Shape fixity (**a**) and shape recovery ratios at T_g_ + 20°C (**b**) of the V-fa/ECO shape-memory CFRP at 40 wt.% CF versus deformation cycles.

Furthermore, the shape-memory cycling performances of the composites were also evaluated. For this experiment, the composites at 40 wt.% were chosen due to their best overall shape fixity and effective shape recovery ratio values. The shape fixity (Figure 8a) and shape recovery (Figure 8b) values of the composite specimens at 0–3 wt.% GnPs experienced a slight decrease in both values after six cycles, but this remained relatively negligible overall. On the other hand, a severe decrease could be observed with the 5 wt.% GnPs specimen after the second cycle. The subsequent shape-memory cycles caused the fiber to repeatedly accumulate fatigue, resulting in increasing numbers of defects forming in the composite structure.

## 4. Conclusions

In this study, a shape-memory poly(V-fa/ECO) composite with carbon fiber reinforcement and a graphene nanoplatelet filler was successfully prepared. The reinforcement and filler addition resulted in a significant improvement in the mechanical properties and shape recovery stress, with the maximum performance being noticed with the optimum values of 40 wt.% CF fiber and 3 wt.% GnPs, where an improvement of 16.8% and 16.2% in terms of the flexural strength and modulus were achieved, respectively. Similarly, the dynamic mechanical properties were also noticeably improved, with the results achieved suggesting a heterogenous network between the ECO and V-fa. However, as the fiber content was increased further, the spring-back became more pronounced, thereby negatively affecting the shape fixity ratio. Nevertheless, a high effective shape recovery ratio (≥90%) was still maintained up until 60 wt.% fiber. In summary, a better shape-memory performance was obtained at a lower fiber content, with the composites fabricated at 40 wt.% CF having a shape fixity and shape recovery ratio in the range of 81–93% and 85–96%, respectively. Moreover, the composites retained a relatively stable shape-memory performance even after six cycles. Overall, the obtained composite suggested a material with improved mechanical and shape-memory properties with more diverse potential applications.

## Figures and Tables

**Table 1:** Flexural properties of neat V-fa/ECO (50/50 wt.%) and V-fa/ECO CFRP.

Sample	GnPs Content (wt.%)	Flexural Strength (MPa)	Flexural Modulus (GPa)
V-fa/ECO	0	27.69 ± 4.71	0.55 ± 0.13
40%CF	0	117.5 ± 5.50	10.3 ± 0.78
	1	123.02 ± 10.04	11.97 ± 1.38
	3	137.21 ± 18.70	12.75 ± 1.87
	5	130.15 ± 12.97	13.56 ± 1.86
50%CF	3	176.61 ± 2.89	21.65 ± 1.77
60%CF	3	181.92 ± 10.12	25.2 ± 3.48
70%CF	3	141.42 ± 11.92	24.8 ± 4.63

**Table 2:** Dynamic mechanical properties of CFRP composites.

Sample	GnPs Content (wt.%)	E′ (GPa) (−100 °C)	E′ (GPa) (T_g_ + 40 °C)	T_g_ (°C)
40%CF	0	20.1	1.71	100
	1	20.9	1.98	102
	3	21.4	1.99	104
	5	20.3	1.55	103
50%CF	3	26.0	4.49	108
60%CF	3	36.4	6.46	112
70%CF	3	36.0	8.68	125

## Data Availability

Data is contained within the article.
